# Novel genetic locus at MHC region for esophageal squamous cell carcinoma in Chinese populations

**DOI:** 10.1371/journal.pone.0177494

**Published:** 2017-05-11

**Authors:** Peng Zhang, Xin-Min Li, Xue-Ke Zhao, Xin Song, Ling Yuan, Fang-Fang Shen, Zong-Min Fan, Li-Dong Wang

**Affiliations:** 1 Henan Key Laboratory for Esophageal Cancer Research, The First Affiliated Hospital of Zhengzhou University, Zhengzhou, Henan, China; 2 Department of Pathology, The Maternal and Child Health Care Hospital of Zhengzhou, Zhengzhou, Henan, China; 3 Department of Radiotherapy, The Affiliated Cancer Hospital of Zhengzhou University, Henan Cancer Hospital, Zhengzhou, Henan, China; 4 The Key Laboratory for Tumor Translational Medicine, The Third Affiliated Hospital of Xinxiang Medical University, Xinxiang, Henan, China; National Institute of Environmental Health Sciences, UNITED STATES

## Abstract

**Background:**

Our previous genome-wide association study (GWAS) identified three independent single nucleotide polymorphisms (SNPs) in human major histocompatibility complex (MHC) region showing association with esophageal squamous cell carcinoma (ESCC). In this study, we increased GWAS sample size on MHC region and performed validation in an independent ESCC cases and normal controls with aim to find additional loci at MHC region showing association with an increased risk to ESCC.

**Methods:**

The 1,077 ESCC cases and 1,733 controls were genotyped using Illumina Human 610-Quad Bead Chip, and 451 cases and 374 controls were genotyped using Illumina Human 660W-Quad Bead Chip. After quality control, the selected SNPs were replicated by TaqMan genotyping assay on another 2,026 ESCC cases and 2,384 normal controls.

**Results:**

By excluding low quality SNPs in primary GWAS screening, we selected 2,533 SNPs in MHC region for association analysis, and identified 5 SNPs with p <10^−4^. Further validation analysis in an independent case-control cohort confirmed one of the 5 SNPs (rs911178) that showed significant association with ESCC. rs911178 (P_GWAS_ = 6.125E-04, OR = 0.644 and P_replication_ = 1.406E-22, OR = 0.489) was located at upstream of *SCAND3*.

**Conclusion:**

The rs911178 (*SCAND3* gene) in MHC region is significantly associated with high risk of ESCC. This study not only reveal the potential role of MHC region for the pathogenesis of ESCC, but also provides important clues for the establishment of tools and methods for screening high risk population of ESCC.

## Introduction

Esophageal cancer (EC) is the sixth most common cancer deaths worldwide and the fourth leading cancer deaths in China. There were an estimated 455,800 new EC patients and 400,200 deaths per year worldwide while 291,238 new incidences and 218,958 mortalities in China [1,2]. Tai-Hang Mountain at Henan, Hebei and Shanxi provinces’ junction are the highest risk area for EC in China. The ESCC and esophageal adenocarcinoma are the two main histological types. More than 90% cases are ESCC in China, compared to about 20% in Western countries [3]. In high-risk regions, known risk factors include nutritional deficiencies, low intake of fresh fruits and vegetables, intake of pickled vegetables, intake of nitrosamine-rich or mycotoxin-contaminated foods, drinking beverages at high temperatures, and low socioeconomic status [4,5]. The dramatic geographic distribution and apparent family aggregation suggest that both environmental and genetic factors may play important roles in pathogenesis of EC [6,7].

Several studies suggest that immune defense mechanism may play an important role in the esophageal carcinogenesis [8]. The human MHC is the most important region for autoimmunity, which encodes human leukocyte antigens (HLA) responsible for antigen presentation to T cells. The HLA gene complex is located on the short arm of chromosome 6 and covers an about 3.5 Mb segment that included three genomic regions, class I (1.9 Mb; HLA-A, HLA-B, and HLA-C), class III (0.7 Mb), and class II (0.9 Mb). The HLA seems to be generated through repeated gene duplication and conversion during evolution [9]. An extended MHC (xMHC) region is densely populated with genes that are critical for innate and adaptive immunity in humans, spanning about 7.6 Mb that covers over 250 known expressed loci. The xMHC is divided into five sub-regions consisting of the extended class I region (3.9 Mb), the classical class I, III, and II clusters, and extended class II region (0.2 Mb) [10–12].

In our previous studies, we report 10 SNP loci and 8 corresponding genes responsible for increased risk of developing ESCC through GWAS in Chinese populations [4,13,14]. We also report an association of 3 independent SNPs in the MHC region with ESCC. However, significance of the 3 MHC risk SNPs remains unknown [15]. To validate the association of the MHC loci, in this study, we analyzed all possible SNPs in MHC region in an increased number of GWAS samples, and performed TaqMan-based genotyping in independent ESCC and normal controls.

## Materials and methods

### Study populations

This study was approved by the ethical review committee of Zhengzhou University and conducted following Declaration of Helsinki principles. All patients and normal controls in this study have provided written informed consent.

Hospital-based ESCC case-control design was used for this study. Subjects of Chinese Han ESCC and normal controls for GWAS scan and replications were recruited from the high-incidence areas for ESCC in northern China and Endoscopic Screening Centers within multiple hospitals for early detection of upper gastrointestinal tumors [4]. The patients were confirmed as ESCC by histopathology and the controls were confirmed without early ESCC and other upper gastrointestinal tumors by upper gastrointestinal endoscopy. The ESCC patients and normal controls were interviewed to obtain demographic and lifestyle histories related to cancer risks. Family histories of ESCC patients regarding upper gastrointestinal cancers in the first-, second-, and third-degree relatives were obtained through questionnaires. All normal controls did not have a family history of cancers.

In the screening phase of GWAS, the previously published 1,077 ESCC patients and 1,733 normal controls of Chinese Han descent were genotyped using Illumina Human610-Quad BeadChip [4]. New cohort of 451 ESCC patients and 374 normal controls were genotyped using the Illumina Human660W-Quad BeadChip. A total of 3,635 samples of Chinese Han descent were screened, including 1,528 ESCC patients (921 male cases, 607 female cases, mean age 61 ± 9 years) and 2,107 controls (1,052 males, 1,055 females, with an average age of 31 ± 15 years) (Table 1).

**Table 1:** Samples used in MHC region GWAS and replication.

	ESCC Cases			Controls		
	n	Mean age (S.D.)	Male/female	n	Mean age (S.D.)	Male/female
ESCC GWAS	1,528	61 (9)	921/ 607	2,107	31 (15)	1,052/ 1,055
TaqMan validation	2,026	60 (9)	1,256/ 770	2,384	50 (11)	1,198/ 1,186

In validation phase, the TaqMan genotyping assays were used for replication in a new separate 4,410 people samples, including 2,026 Chinese Han ESCC cases (1,256 males, 770 females, mean age 60 ± 9 years) and 2,384 normal controls (1,198 males, 1,186 females, with an average age of 50 ± 11 years) (Table 1).

### GWAS screening

Genomic DNAs were extracted from peripheral blood by using FlexiGene DNA kits (Qiagen, Hilden, Germany). The concentration of DNA was normalized to 50 ng/ μl with Nanodrop 2000 Spectrophotometer (Thermo Fisher Scientific, Waltham, USA). 200 ng of DNA were used for genotyping. The genome-wide genotyping analysis were conducted using Illumina Human 610- and 660W- Quad BeadChips (Illumina, San Diego, USA) in the Key Laboratory of Dermatology (Anhui Medical University, Hefei, China).

Each DNA sample had gone through whole-genome amplification, fragmentation, precipitation, and re-suspension in hybridization buffer. Denatured samples were hybridized on prepared Illumina Human 610- or 660W- Quad BeadChips. After hybridization, the Bead Chips oligonucleotides were extended by a single labeled base, which was detected by fluorescence imaging with an Illumina Bead Array Reader. Normalized bead intensity data obtained from each sample were loaded into the Illumina Bead Studio 3.2 software, which converted fluorescence intensities into SNP genotypes. The clustering of genotypes was carried out with Gen-Call 6.2.0.4 software, which assigns a quality score to each locus and an individual genotype confidence score that is based on the distance of a genotype from the center of the nearest cluster.

We performed principal components analysis (PCA) to identify genetic outliers and removed genetically deviated samples using EIGENSTRAT 3.0 software package [16]. To extract the principal components, original script was modified. Criteria [4] for quality control were: 1) Drop if call rate of genotype < 0.90 in the cases or normal controls; 2) Drop if minor allele frequency (MAF)< 0.01 in the cases and normal controls; 3) Drop if the P value of Hardy-Weinberg equilibrium (HWE)< 10^−7^ in the normal controls; 4) All the SNPs on the X, Y and mitochondrial chromosomes as well as the copy number probes were excluded from the statistical analysis; 5) Only shared SNP markers among different Illumina BeadChip were considered. The SNPs in the MHC region potentially associated with high risk of ESCC were selected after removing unqualified SNPs.

### TaqMan replication

Criteria [4] of SNP loci selection for TaqMan-based assay were: 1) the MAF> 0.02 in the cases and controls; 2) the P value of HWE ≥ 0.001 in the controls; 3) the P value of GWAS analysis (Cochran-Armitage trend test) < 10^−4^; 4) the related genes had functional role in carcinogenesis. The high risk SNPs were finally selected from these criteria. For the replication study, DNA concentration was normalized to 15-20ng/μl with Nanodrop 2000 Spectrophotometer. Approximately 15ng of genomic DNA was used to genotype each sample. Genotypes for the selected SNPs were obtained using the TaqMan genotyping assay on 7900HT Fast Real-Time polymerase chain reaction (PCR) system (Applied Biosystems, Foster City, USA) in the Key Laboratory of Dermatology (Anhui Medical University).

### Statistical analysis

The SNP call rates, MAF, and HWE were calculated. Quality controlled genotyping data were analyzed and outputted with Plink1.07 software [17]. Association analyses were performed on ESCC cases and genetically matched controls using the Cochran-Armitage trend test with genomic control correction for population stratification. The P value, odds ratio (OR) and 95% confidence interval (95% CI) were calculated using Cochran-Armitage trend test.

## Results

### Outlier removal and SNP selection

After removing 1,051 deviated samples from controls by the PCA, the genetically matched 1,528 ESCC cases and 1,056 normal controls were remained (genome-wide χ^2^ inflation factor λ_gc_ = 1.07). The genome-wide SNPs scanning were conducted on the DNA of matching samples. After quality control checking, the 488,919 SNPs were left for final analysis, among which 2,533 SNPs were located at MHC region. Further analysis identified 5 SNPs (P_GWAS_ < 10^−4^) in MHC region for validation: rs17533090 (P_GWAS_ = 9.709E-06, OR = 1.503, 95%CI = 1.254–1.802), rs35399661 (P_GWAS_ = 6.070E-06, OR = 1.712, 95%CI = 1.354–2.166), rs1536501 (P_GWAS_ = 8.874E-04, OR = 1.805, 95%CI = 1.268–2.568), rs911178 (P_GWAS_ = 6.125E-04, OR = 0.644, 95%CI = 0.500–0.830) and rs6901869 (P_GWAS_ = 2.523E-05, OR = 1.973, 95%CI = 1.430–2.722) (Table 2).

**Table 2:** Five SNPs were selected for validation.

SNP	Chr.	Position	Gene	effect allele	P_GWAS_	OR(95%CI)	F-A^a^	F-U^b^
rs17533090	6p21.32	32698700	*HLA-DQA1*	T	9.709E-06	1.503(1.254–1.802)	0.132	0.092
rs35399661	6p21.32	32698968		C	6.070E-06	1.712(1.354–2.166)	0.130	0.080
rs1536501	6p21.31	33835863	*LEMD2*	T	8.874E-04	1.805(1.268–2.568)	0.037	0.021
rs911178	6p22.1	28682394	*SCAND3*	T	6.125E-04	0.644(0.500–0.830)	0.040	0.061
rs6901869	6p21.33	31318244	*HLA-C*	A	2.523E-05	1.973(1.430–2.722)	0.047	0.025

^a ^F-A: Minor allele frequency in cases

^b ^F-U: Minor allele frequency in controls

The 5 SNPs were located at the different linkage disequilibrium (LD) blocks of MHC region. rs17533090 and rs35399661 on 6p21.32 fall within a high LD block between *HLA-DQA1* and *HLA-DRB1*. rs1536501 on 6p21.31 is located between LEM domain containing 2 (*LEMD2*) and inositol hexakisphosphate kinase 3 (*IP6K3*). rs911178 on 6p22.1 is located 35-kb upstream of SCAN domain containing 3 (*SCAND3*). rs6901869 on 6p21.33 is located between *HLA-C* genes and HLA complex group 27 (*HCG27*).

### TaqMan-based validation

**Fig 1:**
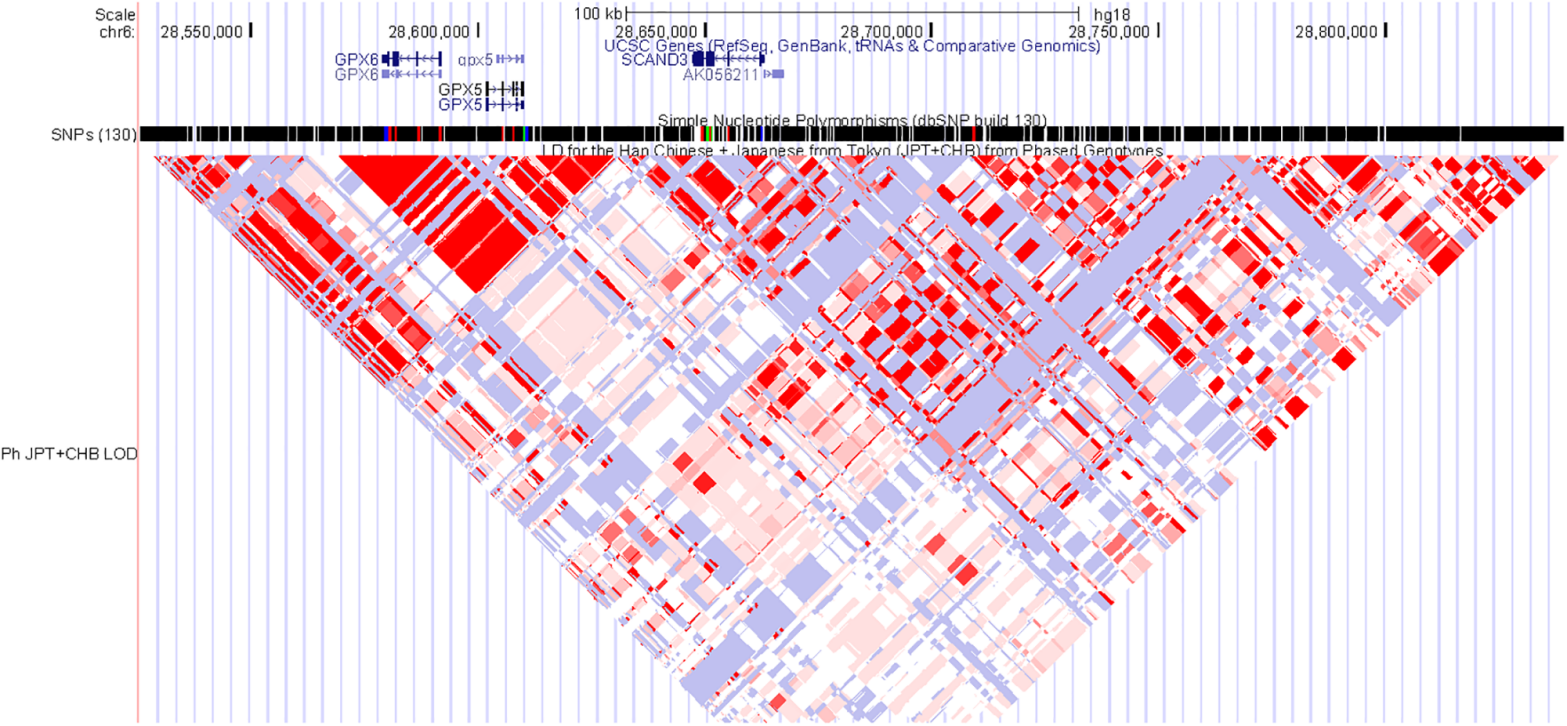
The LD pattern of signal at 6p22.1 in Asian population (CHB+JPT).

The TaqMan validation was conducted on 2,026 cases and 2,384 controls for 5 selected SNPs. The concordance rate among the genotypes from the Illumina and TaqMan analyses was >99%. We checked the cluster patterns of the 5 SNPs from the genotyping data from the Illumina and TaqMan analyses to confirm their good quality. rs17533090 and rs35399661 did not pass the HWE test (P_HWE_ <10^−5^). rs1536501 and rs6901869 did not have significant p value (P_replication_ >0.01). Finally, one SNP rs911178 was validated with P_replication_ = 1.406E-22, OR = 0.489. The SNP is located 35-kb upstream of *SCAND3* on 6p22.1 (Fig 1, Table 3).

**Table 3:** The results of TaqMan validation.

SNP	F-A^a^	F-U^b^	P_HWE_^c^	Call rate	P_replication_	OR	Q	I
rs17533090	0.317	0.135	1.238E-05	0.884	1.634E-75	2.461	0	97.460
rs35399661	0.146	0.100	2.840E-11	0.908	NA	NA	NA	NA
rs1536501	0.027	0.040	0.415	0.974	0.400	0.918	0	95.19
rs911178	0.051	0.110	0.229	0.919	1.406E-22	0.489	0.01	84.94
rs6901869	0.028	0.029	0.262	0.987	0.015	1.282	0.001	91.06

^a ^F-A: Minor allele frequency in cases

^b ^F-U: Minor allele frequency in controls

^c ^P_HWE_: P value of HWE in controls

## Discussion

Several studies have shown that the chromosomal 6p21-6p22 is a hot spot for loss of heterozygosity in ESCC, which results in the downregulation of HLA class I genes [18–20]. Loss of HLA class I and gain of class II protein expression are frequently observed in ESCC. The HLA-DRB1 allele has been correlated with the risk of ESCC. These support the notion that structural variation in the MHC region might be a major mechanism related to genetic susceptibility to ESCC [18–22]. In a joint analysis of NCI, Beijing, and our laboratory we identified that SNP rs35597309 at MHC class II gene region was associated with ESCC [13]. In this study, we identified another important risk locus in MHC region using GWAS, followed by TaqMan validation. The SNP rs911178 is located at upstream of *SCAND3* (also known as *ZBED9* or *ZNF452*). This gene encodes a protein of unknown function with CHCH (C-terminal coiled-coil-helix-coiled-coil-helix motif) and hATC domains (N-terminal hAT family dimerisation motif). It is down-regulated during mouse embryonic stem cell differentiation [23]. *SCAND3* is involved in the self-renewal of mouse embryonic stem cells. SCAN domains are typically associated with transcriptional regulation of gene expression suggests that *SCAND3* is transcription factor [23,24].

GWAS can identify susceptibility loci for cancers by simultaneously comparing hundreds of thousands of SNPs between human genome from cases and healthy individuals. The identified new genetic loci may also further elucidate the factors in the development of cancer. Several GWAS studies not only add to the known genetic factors that predispose individuals to ESCC, but also highlight the importance of genetic factors and genetic heterogeneity in the development of ESCC, which could advance our understanding of the pathogenesis and carcinogenesis of ESCC [25–27].

In summary, we identified and validated that the rs911178 (*SCAND3* gene) in MHC region is significantly associated with the high risk of ESCC through GWAS and TaqMan genotyping assay. Our study provides more understanding of MHC region for the pathogenesis of ESCC, also provides important clues for the establishment of the tools and methods at the screening for high risk population. Further functional studies were needed to elucidate the molecular mechanisms underlying rs911178 on ESCC. Additionally, fine mapping and sequencing in these loci would be required to determine the optimal genetic variants to be studied in laboratory systems to explain these association signals in the future.

## Supporting information

S1 File STROBE_checklist_v4_combined_PlosMedicine. File contains the STROBE Checklist of this paper.
(DOCX)
